# Genetic Analysis, Phenotypic Spectrum and Functional Study of Rare Osteogenesis Imperfecta Caused by *CRTAP* Variants

**DOI:** 10.1210/clinem/dgae025

**Published:** 2024-01-12

**Authors:** Bingna Zhou, Peng Gao, Jing Hu, Xiaoyun Lin, Lei Sun, Qian Zhang, Yan Jiang, Ou Wang, Weibo Xia, Xiaoping Xing, Mei Li

**Affiliations:** Department of Endocrinology, Key Laboratory of Endocrinology, National Health and Family Planning Commission, Peking Union Medical College Hospital, Chinese Academy of Medical Sciences and Peking Union Medical College, Beijing 100730, China; Department of Orthopedics, Peking Union Medical College Hospital, Peking Union Medical College, Chinese Academy of Medical Science, Beijing 100730, China; Department of Endocrinology, Key Laboratory of Endocrinology, National Health and Family Planning Commission, Peking Union Medical College Hospital, Chinese Academy of Medical Sciences and Peking Union Medical College, Beijing 100730, China; Department of Endocrinology, Key Laboratory of Endocrinology, National Health and Family Planning Commission, Peking Union Medical College Hospital, Chinese Academy of Medical Sciences and Peking Union Medical College, Beijing 100730, China; Department of Endocrinology, Key Laboratory of Endocrinology, National Health and Family Planning Commission, Peking Union Medical College Hospital, Chinese Academy of Medical Sciences and Peking Union Medical College, Beijing 100730, China; Department of Endocrinology, Key Laboratory of Endocrinology, National Health and Family Planning Commission, Peking Union Medical College Hospital, Chinese Academy of Medical Sciences and Peking Union Medical College, Beijing 100730, China; Department of Endocrinology, Key Laboratory of Endocrinology, National Health and Family Planning Commission, Peking Union Medical College Hospital, Chinese Academy of Medical Sciences and Peking Union Medical College, Beijing 100730, China; Department of Endocrinology, Key Laboratory of Endocrinology, National Health and Family Planning Commission, Peking Union Medical College Hospital, Chinese Academy of Medical Sciences and Peking Union Medical College, Beijing 100730, China; Department of Endocrinology, Key Laboratory of Endocrinology, National Health and Family Planning Commission, Peking Union Medical College Hospital, Chinese Academy of Medical Sciences and Peking Union Medical College, Beijing 100730, China; Department of Endocrinology, Key Laboratory of Endocrinology, National Health and Family Planning Commission, Peking Union Medical College Hospital, Chinese Academy of Medical Sciences and Peking Union Medical College, Beijing 100730, China; Department of Endocrinology, Key Laboratory of Endocrinology, National Health and Family Planning Commission, Peking Union Medical College Hospital, Chinese Academy of Medical Sciences and Peking Union Medical College, Beijing 100730, China

**Keywords:** osteogenesis imperfecta, novel *CRTAP* variants, pathogenic mechanism

## Abstract

**Objective:**

Deficiency of cartilage-associated protein (CRTAP) can cause extremely rare autosomal recessive osteogenesis imperfecta (OI) type VII. We investigated the pathogenic mechanisms of *CRTAP* variants through functional studies on bones of patients with OI.

**Methods:**

Two nonconsanguineous families with *CRTAP* mutations were included and their phenotypes and genotypes were evaluated. Bone specimens were obtained from 1 patient with OI and a normal control during orthopedic surgery. The impacts of the novel variant on the *CRTAP* transcript were confirmed. The expression levels of *CRTAP* mRNA and CRTAP protein were analyzed. The quantification of prolyl 3-hydroxylation in the α1 chain of type I collagen was evaluated.

**Results:**

Patients with OI type VII had early-onset recurrent fractures, severe osteoporosis, and bone deformities. The c.621 + 1G > A and c.1153-3C > G mutations were identified in *CRTAP* in the patients with OI. The c.621 + 1G > A variant was a novel mutation that could impair mRNA transcription, leading to a truncated CRTAP protein. In a patient with c.621 + 1G > A and c.1153-3C > G mutations in *CRTAP*, the mRNA and protein levels of *CRTAP* in osteoblasts were significantly decreased and the osteoid volume and osteoblast numbers were markedly reduced compared with those in the normal control individual. This was simultaneously accompanied by significantly reduced prolyl 3-hydroxylation at Pro986 in the α1 chain of type I collagen and invisible active bone formation in bone.

**Conclusion:**

The novel c.621 + 1G > A mutation in *CRTAP* expands the genotypic spectrum of type VII OI. Biallelic mutations of c.621 + 1G > A and c.1153-3C > G in *CRTAP* can lead to reduced *CRTAP* mRNA and deficient CRTAP protein in osteoblasts, which reduces 3-hydroxylation in Pro986 of the α1 chain of type I collagen and impairs bone formation, thus contributing to severe OI type VII.

Osteogenesis imperfecta (OI) is a heritable multisystem disorder characterized by low bone mass and impaired bone quality, resulting in recurrent fractures, progressive skeletal deformities, short statures, and extraskeletal manifestations (1). Several pathogenic genes of OI have been identified in past years, of which mutations in the gene encoding type I collagen (*COL1A1* and *COL1A2*), the major structural protein of bone and skin, cause most cases of OI (2). Mutations in *COL1A1* or *COL1A2* lead to autosomal dominant OI through inducing quantitative deficiencies or structural defects of type I collagen (3). The remaining OI cases are caused by mutations in a variety of other genes, and patients with the recessive form of OI usually show more severe phenotypes, including more fractures, more severe skeletal deformities, shorter stature, and poorer mobility (4).

In 2006, defects in cartilage-associated protein (CRTAP) were identified to cause rare autosomal recessive OI (OMIM #610682) (5). CRTAP is a component of the prolyl-3-hydroxylation complex, which is responsible for posttranslational modification and processing of type I procollagen (6). The complex consists of CRTAP (encoded by *CRTAP*), prolyl 3-hydroxylase 1 (encoded by *LEPRE1*) and cyclophilin B (encoded by *PPIB*) and is located in the rough endoplasmic reticulum (ER). It not only plays an important role as a collagen chaperone but also 3-hydroxylates a single proline residue at position 986 of the α1 chain of collagen type I (5). The human *CRTAP* gene has been mapped to chromosome 3p22.3, and its inactivating mutations lead to severe OI with the phenotypes of multiple fractures, short limbs, and severe osteoporosis recognized at birth or in utero (6). Rhizomelia, wormian bone, soft calvarium, scoliosis, and popcorn epiphysis are also observed in patients with OI type VII (6). The symptom and severity of OI type VII are highly heterogeneous from case to case. However, a full understanding of the skeletal disease associated with the *CRTAP* mutation was hampered by its extremely low incidence, genotypic heterogeneity, and phenotypic diversity.

Therefore, we investigated the genetic analysis and phenotypic spectrum of OI type VII. More importantly, we completed a series of functional studies on patient-derived bones to reveal the pathogenic mechanism of *CRTAP* inactivating mutations causing a rare recessive form of OI.

## Materials and Methods

### Subjects

Two Chinese patients with multiple fractures occurring under minor force were included from different nonconsanguineous families. These patients were clinically suspected to have OI in the endocrinology department of Peking Union Medical College Hospital (PUMCH) between 2018 and 2021. Patient 1 was a 6-year-old girl, who was the only child of her family. She was born full-term with a birth weight of 3250 g and a body length of 50 cm. At 4 months old, she experienced the first fracture at right femur. Subsequently, she suffered a recurring right femoral fracture at the age of 1.5 years old. Her development milestones and intellectual abilities were normal and there was no relevant family history. She came to PUMCH aged 2 years. Patient 2 was a 33-year-old man whose birth status was unknown. He suffered the first bone fracture at 6 months old. Subsequently, more than 10 fragility fractures occurred at the bilateral humerus, femurs, tibia, and fibula, which led to lower limb-bending deformity, muscle atrophy, and wheelchair dependency. The family history was noncontributory. He came to PUMCH aged 30 years.

The study protocol was approved by the scientific ethics committee of PUMCH (ethics committee approval code: JS-2081). The patients or their legal guardians signed informed consent before participation in this study.

### Evaluation of Phenotypes

After obtaining a detailed medical history and family history, a physical examination was conducted. Height and weight were measured using a Harpenden stadiometer (Seritex Inc.), and the data were converted to age- and sex-specific *Z* scores according to growth reference data for normal Chinese children (7).

Fasting blood samples were collected between 8:00 Am and 10:00 Am. An automated Roche electrochemiluminescence system (Roche Diagnostics, Switzerland) was used to detect serum concentrations of various markers including PTH, and 25 hydroxyvitamin D (25OHD), cross-linked C-telopeptide of type I collagen (β-CTX, a bone resorption marker), and procollagen type 1 amino-terminal peptide (P1NP, a bone formation marker). Additionally, calcium (Ca), phosphate (P), alkaline phosphatase (ALP, a marker of bone formation), alanine aminotransferase, and creatinine were detected using an automatic biochemical analyzer in the central clinical laboratory of PUMCH.

Dual-energy X-ray absorptiometry (Lunar Prodigy, GE Healthcare, Madison, WI, USA) was used to measure bone mineral density (BMD) at lumbar spine 1-4, femoral neck (FN), trochanter, and total hip at baseline and follow-up. To exclude the influence of age on BMD, The *Z* scores of BMD were calculated according to the age- and sex-matched normal ranges of Chinese or Asian children (8, 9). Radiographs of the spine, pelvis, and limbs were obtained at baseline and follow-up. Based on the patients' medical history, new fractures were suspected and later confirmed by radiographs.

### Treatment and Follow-up

Patient 1 received an annual IV infusion of 2.5 mg zoledronic acid (Aclasta, Novartis Pharmaceuticals) for 3 years because her body weight was less than 25 kg (10). She also received supplementation with 300 mg calcium daily and 0.25 μg calcitriol every other day (10). Patient 2 received an IV infusion with 5 mg zoledronic acid annually for 2 years. He underwent right lower-limb orthopedic surgery at PUMCH to correct severe curvature deformities in 2021. Because radiographs showed a significant delay in fracture healing after surgery, 20 µg of teriparatide (Forsteo, Lilly France) was subcutaneously injected daily for 7 months. After surgical cuff healing, he switched to receiving subcutaneous injection of 60 mg of denosumab (Prolia, Amgen) every 6 months. After a year of denosumab treatment, he underwent left lower-limb orthopedic surgery in PUMCH in 2023. Because the healing of fractures was delayed, teriparatide was used again to accelerate the healing of fractures. He took 600 mg of calcium and 0.25 μg of calcitriol on a daily basis.

### Histological Analysis of Tibia

The tibial bone specimens were surgically obtained from patient 2 during orthopedic surgery. Specifically, the samples were collected from the distal third of the left tibial diaphysis. We also collected tibial bone specimens from an age- and sex-matched normal man who underwent surgery for a violent tibial fracture from the distal third of the right tibial diaphysis (Supplementary Fig. S1) (11).

After collecting the bone samples, they were immediately fixed and preserved in 70% ethanol. Then, they were embedded in modified methyl methacrylate without being decalcified. Sections of 10 µm in thickness were cut using a microtome (Leica RM2016, Leica Microsystems) and deplasticized; the resin was removed before staining. Toluidine blue staining (Servicebio, catalog No. GP1052) was used to stain the bone sections to view the numbers of osteoblasts and osteoclasts; Goldner trichrome staining (Servicebio, catalog No. G1064) was used to identify osteoid and mature bone.

### Detection of Pathogenic Mutations

A QIAamp DNA Mini Kit (Qiagen) was used to extract the genomic DNA from the peripheral leukocytes of the 2 OI patients and their family members. According to the clinical utility gene card for osteogenesis imperfecta (12), we first performed a sequence analysis of the *COL1A1* and *COL1A2* genes on genomic DNA. If no causative variant was identified, the next step was to complete quantitative real-time PCR (qPCR), multiplex ligation-dependent probe amplification, or array-based analysis to determine whether a deletion or duplication of some or all of the coding regions of either gene had occurred. If no mutation was identified, genomic DNA was sequenced using a targeted next-generation sequencing (NGS) panel (Illumina HiSeq 2000 platform, Illumina, Inc., San Diego, CA, USA) to detect mutations in the recessive genes of OI according to the previously described protocol (10). Bioinformatic analysis was carried out following previously reported pipelines (13). The mutations identified by NGS were subsequently confirmed by PCR and Sanger sequencing with primers designed using Primer 3 (http://bioinfo.ut.ee/primer3-0.4.0/) (13). Primer sequences of *CRTAP* are shown in Supplementary Table S1 (*CRTAP*-IVS2-F/R and *CRTAP*-IVS6-F/R) (11). The pathogenicity of the variants was assessed according to the 2015 American College of Medical Genetics and Genomics and Association for Molecular Pathology (ACMG/AMP) Standards and Guidelines (14).

### Assessment of *CRTAP* Transcripts

An in vitro minigene splicing assay was carried out to evaluate the impact of the novel c.621 + 1G > A variant of *CRTAP*. The minigene plasmid was designed to be inserted between exons 1 and 3 of *CRTAP*. The primer sequences of amplified genomic DNA are shown in Supplementary Table S1 (*CRTAP*-AF/R and *CRTAP*-BF/R) (11). The amplified products were cloned and inserted into the pMini-CopGFP vector (Supplementary Fig. S2A, Hitrobio Biotechnology, Beijing, China) (11) and digested with restriction enzymes 5′-BamHI/3′-XhoI using a ClonExpress II One Step Cloning Kit (Vazyme, Nanjing, China). The mutant plasmid was generated by site-directed mutagenesis of the wild-type plasmid using *CRTAP*-MT-F/R primers (Supplementary Table S1) (11). Supplementary Fig. S2B (11) shows a schematic diagram of the minigene construction. The wild-type and variant minigene plasmids were transiently transfected into human embryonic kidney 293 T cells using Lipofectamine 2000 (Invitrogen, Carlsbad, CA, USA). Subsequently, transfected cells were harvested and total cellular RNA was isolated with TRIzol reagent (Ambion, Auckland, New Zealand). Then, RT-PCR and Sanger sequencing were performed to detect changes in mRNA expression using primers for RT-PCR (*CRTAP*-RT-F/R) (Supplementary Table S1) (11). Finally, to analyze the impact of mutations on the protein sequence, the ExPASy Translate tool was used to translate nucleotide sequences into protein sequences.

### Transcriptional Expression Analysis of *CRTAP* in Osteoblasts

Quantitative PCR was used to evaluate the expression of *CRTAP* in osteoblasts (13). The bone specimens of patient 2 and the normal control specimens were cut into fragments, which were washed and digested with 0.25% Trypsin-EDTA (Gibco). Then, the fragments were washed again and digested with 1 mg/mL type II collagenase (Gibco) overnight, α-MEM supplemented with 20% fetal bovine serum was used to stop digestion and then they were centrifuged. After centrifugation, the bone fragments were washed again. The washing solution was discarded and then the precipitation was resuspended and filtered. Osteoblasts were cultured in α-MEM supplemented with 10% fetal bovine serum and 1% pen-strep. Total RNA was extracted from osteoblasts, followed by complementary DNA synthesis. The expression level of *CRTAP* was quantified by qPCR on a Viia 7 Real-Time PCR System (Life Technologies). Primers are shown in Supplementary Table S1 (*CRTAP*-qPCR-F/R and *ACTB*-qPCR-F/R) (11). Relative messenger RNA expression levels were calculated using the 2^−ΔΔCT^ method and normalized to the internal control β-Actin (*ACTB*).

### Protein Expression Analysis of CRTAP in Osteoblasts

Western blotting was used to analyze the protein expression levels of CRTAP in cultured osteoblasts (13). Osteoblasts were lysed in radioimmunoprecipitation assay buffer containing PMSF and Halt Protease and Phosphatase Inhibitor cocktail to extract the protein. The BCA Protein Assay Kit (Thermo Fisher Scientific) was used to quantitate the protein from each cell lysate. Protein was loaded onto a 10% SDS-polyacrylamide gel for electrophoresis and then transferred to poly (vinylidene fluoride) membranes. After being blocked with 5% bovine serum albumin, the membranes were incubated with a primary antibody, mouse anti-human CRTAP monoclonal antibody (Abnova Cat# H00010491-M01, RRID:AB_565612, 1:1000 dilution), and mouse monoclonal to beta actin antibody (Abcam Cat# ab8226, RRID:AB_306371, 1:1000 dilution) and then washed, followed by incubation with a horseradish peroxidase-conjugated secondary antibody (Abcam Cat# ab205719, RRID:AB_2755049, 1:5000 dilution). Finally, the immunoblots were visualized by enhanced chemiluminescence.

### Liquid Chromatography-Tandem Mass Spectrometry Analysis

Liquid chromatography-tandem mass spectrometry analysis was performed on tryptic peptides of the α1 chain of type I collagen to evaluate the effects of *CRTAP* mutations on prolyl 3-hydroxylation. For patient 2 and the normal control samples, the α1 chain of type I collagen was cut from SDS-PAGE gels and subjected to in-gel trypsin digestion. Nano liquid chromatography was performed using Easy-nLC 1200 (Thermo Fisher Scientific) by a 150 μm × 15 cm in-house column packed with Acclaim PepMap RPLC C18 (3 μm, 100 Å, Dr. Maisch GmbH, Germany). Mass spectrometry was performed on the tryptic peptides using a Q Exactive Plus Hybrid Quadrupole-Orbitrap Mass Spectrometer (Thermo Fisher Scientific). The raw mass spectrometry files were analyzed and searched against the target protein database using Byonic.

## Results

### Patient Phenotypic Characteristics

**Figure 1.**
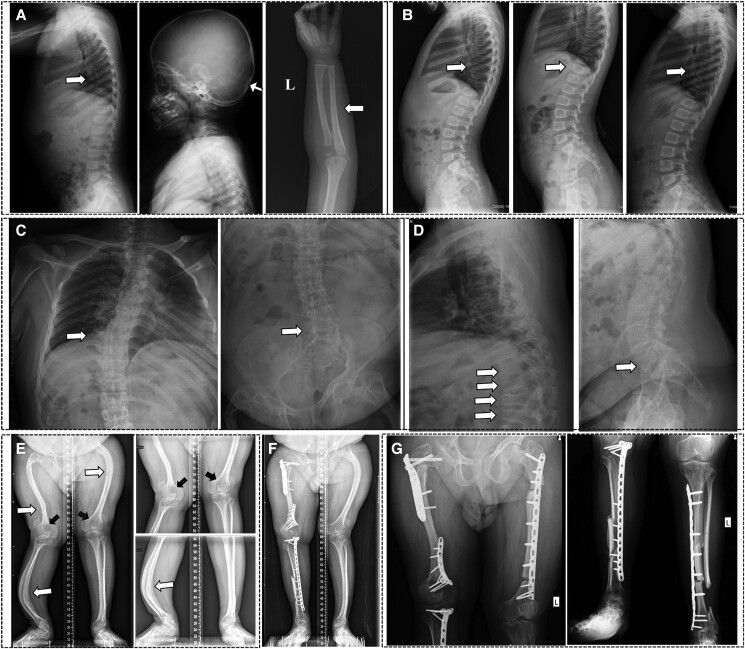
Radiological findings of the patients with *CRTAP* mutations. (A) Radiographs of thoracolumbar and skull and left ulna and radius bone of patient 1 at baseline. (B) Radiographs of thoracolumbar of patient 1 after 1 year, 3 years of zoledronic acid treatment and after discontinuation of zoledronic acid for 1 year. (C) Anteroposterior view of thoracic and lumbar vertebra of patient 2 at baseline. (D) Lateral film of thoracic and lumbar vertebra of patient 2 at baseline. (E) Radiographs of lower extremities of patient 2 at baseline. (F) Radiographs of lower extremities of patient 2 at 5 months after the first surgery. (G) Radiographs of bilateral femurs, tibia, and fibula of patient 2 at 5 months after the second surgery.

Patient 1 experienced recurrent femoral fractures. No scoliosis, hearing loss, blue sclera, or dentinogenesis imperfecta was observed in this patient. Generalized osteoporosis, occipital wormian bone, and slender long bone with thin cortical bone was observed in radiographs (Fig. 1A). Lumbar spine and FN BMD were lower than those of healthy age- and sex-matched control individuals. Serum levels of Ca, P, ALP, β-CTX, P1NP, PTH, and 25OHD were all within age-matched normal ranges. Zoledronic acid treatment increased BMD, and no new fractures occurred during 3 years of treatment. Vertebral body reshaping was observed after zoledronic acid treatment in thoracolumbar radiographs (Fig. 1B).

Patient 2 had more than 10 fractures at multiple sites, leading to severe lower limb-bending deformity, atrophic muscles, movement restriction, and short stature (body height of 133 cm, which is lower than the third percentile of a normal man). He had no extraskeletal manifestations of OI. Radiographs showed severe osteoporosis, scoliosis, multiple vertebral compression fractures (Fig. 1C and 1D), “popcorn” epiphysis, metaphyseal enlargement of distal femurs, and deformities of bilateral femurs, tibia, and fibula (Fig. 1E). Lumbar spine BMD measurement was interfered by severe scoliosis and multiple vertebral compression fractures. The FN BMD was markedly lower than that of the healthy control individuals. He had normal Ca, ALP, β-CTX, P1NP, and PTH levels and low 25OHD levels (18.6 ng/mL). After sequential treatment with zoledronic acid, teriparatide, and denosumab, the FN BMD was significantly increased. He received lower limb orthopedic surgery at PUMCH, and the bilateral limb deformity was significantly improved (Fig. 1F and 1G). The clinical phenotypes of the patients are summarized in Table 1.

**Table 1.** Clinical characteristics of patients with *CRTAP* mutations

	Patient 1	Patient 2	Reference range
Gender	Female	Male	
Disease severity	Moderate	Severe	
Sillence classification	IV	III	
Family history	No	No	
Age of initiating fracture (y)	0.33	0.5	
Age at first diagnosis (y)	2	30	
Peripheral fracture, times	2	10+	
Fracture (locations)	Right femur	Bilateral humerus, femurs, tibia, and fibula	
Multiple vertebral fractures	No	Yes	
Scoliosis	No	Yes	
Limb deformities	No	Yes	
Blue sclera	No	No	
Dentinogenesis imperfecta	No	No	
Hearing loss	No	No	
Intellectual development	Normal	Normal	
Height (cm)/height *Z* score	83/−1.2	133/−6.6	
Weight (kg)/weight *Z* score	13/1.0	65/0.3	
ALT (U/L)	19	39	9-50
Cr (μmol/L)	27	58	45-84
ALP (IU/L)	291	122	Adults: 45-125; children: 42-390
Ca (mmol/L)	2.58	2.36	2.13-2.70
P (mmol/L)	1.67	0.70	Adults: 0.81-1.45; children: 0.95-2.65
β-CTX (ng/mL)	1.11	0.41	Adults: 0.21-0.44; children: 0.40-3.30
P1NP (ng/mL)	988.3	41.3	Adults: 15.1-58.6; children: 30.0-3000
PTH (pg/mL)	15.3	60.1	15.0-65.0
25OHD (ng/mL)	50.3	18.6	Deficiency: < 20; Insufficiency: 20-30; sufficiency: >30
LS BMD (g/cm^2^)	0.459	/*^a^*	2-year-old girl: 0.529 ± 0.085 30-year-old man: 1.180 ± 0.140
LS BMD *Z* score	−0.8	/*^a^*	>−2.0
FN BMD (g/cm^2^)	0.393	0.587	2-year-old girl: 0.576 ± 0.056 30-year-old man: 1.037 ± 0.200
FN BMD *Z* score	−3.3	−3.5	>−2.0
TR BMD (g/cm^2^)	0.348	0.694	/
TH BMD (g/cm^2^)	0.560	0.901	/

Abbreviations: 25OHD, 25-hydroxyvitamin D; ALT, alanine aminotransferase; ALP, alkaline phosphatase; BMD, bone mineral density; β-CTX, cross-linked C-telopeptide of type I collagen; Ca, serum calcium; Cr, creatinine; FN, femoral neck; LS, lumbar spine; P, serum phosphate; P1NP, procollagen type 1 amino-terminal peptide; TR, trochanter; TH, total hip.

^*a*^LS BMD measurement and its *Z* score was interfered with by severe scoliosis and multiple vertebral compression fractures.

### *CRTAP* Mutations of the Patients

Initially, no causative variants were detected in *COL1A1* and *COL1A2* genes of the 2 patients. Subsequently, no deletions or duplications were observed in any or all of the coding regions of either *COL1A1* or *COL1A2* gene. Following this, the NGS panel revealed *CRTAP* mutations in both patients.

**Figure 2.**
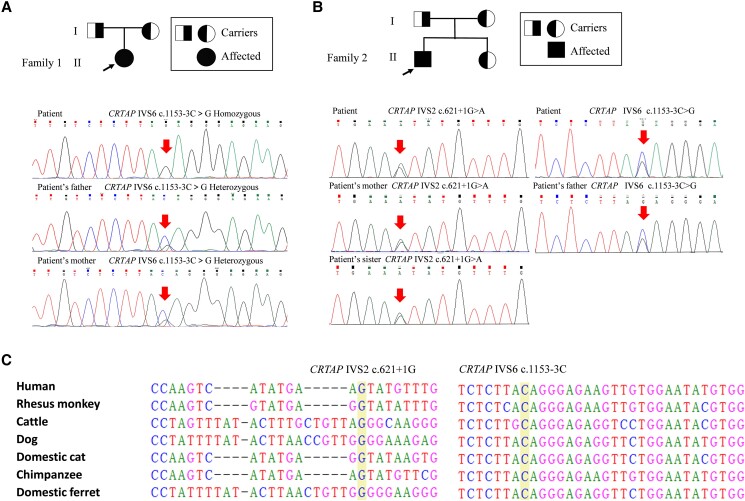
Pedigree and Sanger sequencing of *CRTAP* mutations. (A) Pedigree and Sanger sequencing of family 1 with *CRTAP* mutations. The affected gene site is marked with arrows. (B) Pedigree and Sanger sequencing of family 2 with *CRTAP* mutations. The affected gene sites are marked with arrows. (C) Multiple alignments showed conservation of the mutation sites in *CRTAP* gene in multiple species.

In patient 1, a homozygous mutation of c.1153-3C > G was identified 3 bases upstream of exon 7 of *CRTAP*, and the patient's parents were asymptomatic heterozygous carriers of this variant (Fig. 2A). A previous study indicated that c.1153-3C > G resulted in a broken splice site and intron 6 retention and generated a premature termination codon (15). According to the ACMG/AMP Standards and Guidelines, this mutation was pathogenic (PS3 + PM2 + PM3_Strong + PP1 + PP4).

In patient 2, a novel variant of c.621 + 1G > A at 1 base downstream of exon 2 and the same variant of *CRTAP*, c.1153-3C > G, were identified. The patient's mother and sister carried the c.621 + 1G > A mutation, and his father carried the c.1153-3C > G mutation (Fig. 2B). According to the ACMG/AMP Standards and Guidelines, the c.621 + 1G > A mutation was defined as likely pathogenic (PVS1_Strong + PM2 + PM3_Supporting). Multiple alignments of the 2 mutation sites of *CRTAP* showed conservation of the 2 mutation sites in the *CRTAP* gene in multiple species (Fig. 2C).

### Bone Histology Findings of Patient 2

**Figure 3.**
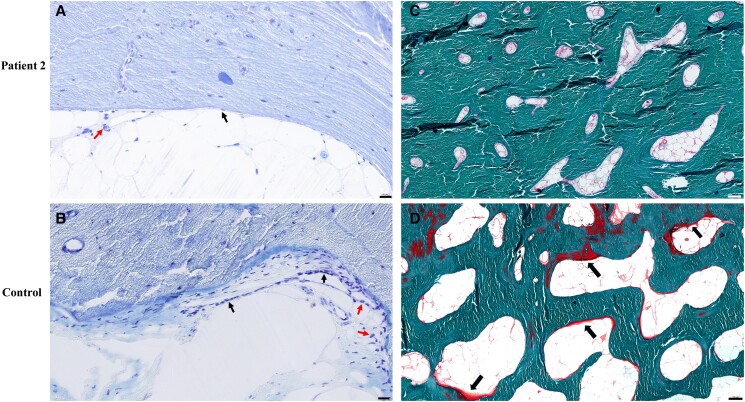
Bone histological characteristics of patient 2 and the control. (A) Toluidine blue staining of tibia of patient 2 40×, scale bar = 20 μm. The underlying cement line was smooth. Few osteoclasts and few osteoblasts (indicated by arrows) were found on the bone surfaces, indicating a low bone formation in patient 2. (B) Toluidine blue staining of tibia of the control 40×, scale bar = 20 μm. The underlying cement line was scalloped. Several multinucleated osteoclasts and multiple osteoblasts (indicated by arrows) were found on the bone surfaces. (C) Goldner trichrome staining of tibia of patient 2 10×, scale bar = 100 μm. Osteoid was stained in red. Osteoid was decreased and active bone formation was invisible in tibial bone slices of patient 2. (D) Goldner trichrome staining of tibia of the control 40×, scale bar = 100 μm. Osteoid was stained in red (indicated by arrows).

In bone histology, a significant decrease in the number of osteoblasts was found in patient 2 compared with the healthy control individual, indicating low bone turnover status. This phenomenon may be related to pathogenic *CRTAP* mutations, but the impact of antiosteoporotic treatment could not be excluded (Fig. 3A and 3B). Moreover, in comparison with the healthy control individual, Goldner trichrome staining of the tibia showed that the volume of osteoid was decreased, and active progression of bone formation was almost undetectable in tibial bone slices of patient 2 (Fig. 3C and 3D).

### Minigene Testing Findings

**Figure 4.**
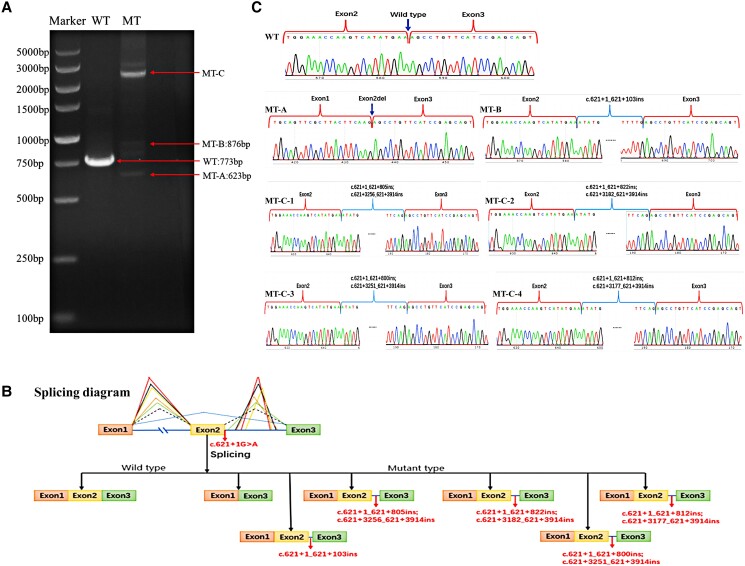
Splicing analysis using a minigene splicing assay. (A) Electrophoretogram of RT-PCR products of the WT and MT plasmids. The WT plasmid had 1 product after RT-PCR; the MT plasmid had 3 products after RT-PCR (MT-A, MT-B, MT-C). (B) Splicing diagram of normal and c. 621 + 1G > A variant-related abnormal splicing. The transcribed mRNA sequence from the wild-type plasmid contained complete exon 1 to 3. The variant plasmid transcribed six mRNA products (MT-A, MT-B, MT-C-1, MT-C-2, MT-C-3, and MT-C-4). (C) Sanger sequencing of WT and 6 mutant transcribed mRNA products. The Sanger sequence of the wild-type plasmid contained complete exon 1 to 3. The Sanger sequence of the MT-A was expressed as NM_006371.4: c.472_621del (p.Ala158_Glu207del). The Sanger sequence of the MT-B was expressed as NM_006371.4: c.621 + 1_621 + 103ins (p.Ser208IlefsTer16). The Sanger sequence of the MT-C-1 was expressed as NM_006371.4: c.621 + 1_621 + 805ins; c.621 + 3256_621 + 3914ins (p.Ser208IlefsTer16). The Sanger sequence of the MT-C-2 was expressed as NM_006371.4: c.621 + 1_621 + 822ins; c.621 + 3182_621 + 3914ins (p.Ser208IlefsTer16). The Sanger sequence of the MT-C-3 was expressed as NM_006371.4: c.621 + 1_621 + 800ins; c.621 + 3251_621 + 3914ins (p.Ser208IlefsTer16). The Sanger sequence of the MT-C-4 was expressed as NM_006371.4; c.621 + 1_621 + 812ins; c.621 + 3177_621 + 3914ins (p.Ser208IlefsTer16). MT, mutant; RT-PCR: reverse transcription-PCR; WT, wild-type.

The pathogenicity of the novel c.621 + 1G > A variant was explored by an in vitro minigene splicing assay, which indicated that c.621 + 1G > A disrupted the splice donor site within intron 2 and was predicted to form a truncated CRTAP protein. As shown in Fig. 4, the transcribed mRNA sequence from the wild-type plasmid contained complete exons 1 to 3, but the variant plasmid transcribed 6 mRNA products (MT-A, MT-B, MT-C-1, MT-C-2, MT-C-3, and MT-C-4). MT-A caused a complete deletion of 150 bp in exon 2 (exon 2 skipping), and the mRNA was expressed as NM_006371.4: c.472_621del (p.Ala158_Glu207del), which may lead to an amino acid deletion to form a truncated protein. MT-B induced a retention of 103 bp in intron 2, and the mutant mRNA was expressed as NM_006371.4: c.621 + 1_621 + 103ins (p.Ser208IlefsTer16), which was predicted to cause a frameshift and form a truncated protein. MT-C transcribed 4 mRNA products, which induced retentions in exon 2. They were predicted to cause frameshifts and form a truncated CRTAP protein (Table 2).

**Table 2.** The changes of mRNA expression and the amino acid in *CRTAP* c.621 + 1G > A in minigene assay

Number	mRNA products	Change of mRNA expression	Amino acid change
1	MT-A	NM_006371.4: c.472_621del	p.Ala158_Glu207del
2	MT-B	NM_006371.4: c.621 + 1_621 + 103ins	p.Ser208IlefsTer16
3	MT-C-1	NM_006371.4: c.621 + 1_621 + 805ins; c.621 + 3256_621 + 3914ins	p.Ser208IlefsTer16
4	MT-C-2	NM_006371.4: c.621 + 1_621 + 822ins; c.621 + 3182_621 + 3914ins	p.Ser208IlefsTer16
5	MT-C-3	NM_006371.4: c.621 + 1_621 + 800ins; c.621 + 3251_621 + 3914ins	p.Ser208IlefsTer16
6	MT-C-4	NM_006371.4: c.621 + 1_621 + 812ins; c.621 + 3177_621 + 3914ins	p.Ser208IlefsTer16

### Expression of CRTAP mRNA and Protein in Osteoblasts

**Figure 5.**
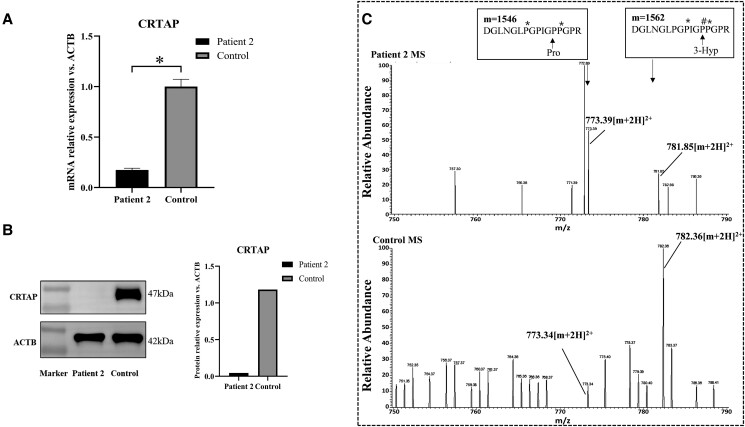
Functional study of *CRTAP* mutations. (A) Relative expression of *CRTAP* mRNA in the osteoblasts from patient 2 and the control. *CRTAP* mRNA level was normalized to the *ACTB* mRNA level and then to the *CRTAP* mRNA level in the control osteoblast line (which was arbitrarily set at 1). T bars represent SDs. (B) Relative expression of CRTAP protein in the osteoblasts from patient 2 and the control. CRTAP protein level was normalized to the ACTB protein level. (C) Mass spectrometric analyses shows decreased 3-hydroxyproline of α1(I) collagen of patient 2 compared with the control. The top boxes represented the masses (m, in atomic mass units) and the amino acid sequences (capital letters; P# and P* denote 3- and 4-hydroxyproline [Hyp], respectively) of collagen peptides that have either undergone 3-hydroxylation at the proline at position 986 (Pro986) (right box, in which the proline is converted to 3-Hyp) or have not (left box). Peptide ions that have undergone normal Pro986 hydroxylation have an m/z ratio of approximately 782, whereas peptides that have not have an m/z ratio of approximately 774.

According to qPCR, patient 2 had only 17.5% of the normal level of the *CRTAP* mRNA transcript in osteoblasts, suggesting a significant decrease in *CRTAP* transcripts compared with the healthy control individual (Fig. 5A). Moreover, as determined by Western blotting, patient 2 had virtually no CRTAP protein expression, indicating that CRTAP protein levels were significantly deficient in the osteoblasts compared with the normal control individual (Fig. 5B).

### Effects of CRTAP Deficiency on the Metabolism of Type I Collagen

Figure 5C shows the cumulative, full scan across the liquid chromatography peak. Peptide ions that had undergone normal Pro986 hydroxylation had an m/z ratio of approximately 782, whereas peptides without normal Pro986 hydroxylation had an m/z ratio of approximately 774. Tandem mass spectrometry analysis revealed that patient 2 had only approximately 32% 3-hydroxylation of α1(I) Pro986 residues, whereas the control had more than 90% α1(I) Pro986 residues. These findings indicate that prolyl 3-hydroxylation at Pro986 in the α1 chain of type I collagen was significantly reduced in patient 2.

## Discussion

In this study, we examined the phenotypic, genotypic, and histological characteristics and pathogenic mechanisms of patients with OI caused by *CRTAP* mutations. We found that early onset, multiple fractures, progressive skeletal deformity, and very low BMD were prominent phenotypic features of patients with type VII OI. The novel mutation of c.621 + 1G > A was identified in exon 2 of *CRTAP*. This mutation could impact the transcription of *CRTAP* mRNA, leading to a truncated CRTAP protein. In patients carrying compound heterozygous mutations of c.621 + 1G > A and c.1153-3C > G in *CRTAP*, the *CRTAP* transcript level was significantly reduced and CRTAP protein was deficient in osteoblasts, which would lead to severely reduced Pro986 hydroxylation in the α1 chain of type I collagen. As a recessive, severe, nonlethal form of OI, patients with *CRTAP* mutations had distinctive histologic characteristics of the bone, including defective osteoid formation and low bone formation.

As shown in previous studies, OI patients with biallelic variants often had severe phenotypes (4). OI type VII is an autosomal recessive form of OI caused by *CRTAP* mutations, which can lead to loss of the prolyl-3-hydroxylase complex and disturb the architecture of the type I collagen structure (6). A recent study summarized the phenotypes of 30 probands with OI type VII (6) and suggested a phenotypic spectrum of multiple fractures at birth or gestational age, shortened extremities, and severe osteoporosis (5, 6, 15-29). Patients with OI type VII often have unique imaging manifestations, including rhizomelia, wormian bone, soft calvarium, scoliosis, and popcorn epiphysis (6). The phenotypes of our patients were consistent with those of previous studies (5, 16-22).

In this study, a known mutation of c.1153-3C > G of *CRTAP* and an unreported mutation of c.621 + 1G > A in exon 2 of *CRTAP* were identified, which expands the genotypic spectrum of type VII OI. The novel c.621 + 1G > A mutation was predicted to be damaging by SpliceAI, dbscSNV_RF, and dbscSNV_ADA. For the first time, we confirmed that the c.621 + 1G > A mutation in *CRTAP* could impact mRNA transcription through minigene testing, thus leading to the formation of a truncated CRTAP protein. Previous studies have shown that the expression and function of the mutant CRTAP protein are impaired in patients with OI type VII (17). Another study showed that mutant *CRTAP* transcripts were detectable, whereas CRTAP protein was undetectable in proband fibroblasts. This triggered a critical level of proteotoxicity that led to senescent death of the proband cells (6). Consistently, we detected reduced *CRTAP* mRNA transcript levels, but virtually no CRTAP protein was observed in the osteoblasts of patients with OI.

CRTAP acts as part of a collagen 3-hydroxylation complex and may also directly mediate collagen helical assembly (30). In this complex, CRTAP plays the role of a helper protein, whereas prolyl 3-hydroxylase 1 provides enzymatic activity for the modification (30). Prolyl 3-hydroxylase, located in the ER, modifies Pro-4Hyp-Gly, specifically the α1(I) Pro986 residue; 3-hydroxylation of α1(I) Pro986 is critical for the structural integrity of type I collagen fibers (5, 30, 31). In cultured fibroblasts and osteoblasts of patients with type VII OI, 3-hydroxylation of the α1 Pro986 residue of type I collagen was significantly reduced (17, 18). A previous study demonstrated that mutations in the prolyl3-hydroxylation complex promoted the synthesis of overmodified collagen and led to the disruption of ER homeostasis (32). All studies emphasize the importance of 3-hydroxylation of the proline of type I collagen. We not only observed reduced prolyl 3-hydroxylation of type I collagen induced by CRTAP deficiency in patients with OI but also found significantly reduced bone formation and less osteoid volume, which reveals the pathological mechanism by which *CRTAP* mutations lead to severe OI.

Currently, bisphosphonates are the main drug for treating bone fragility, but their efficacy in autosomal recessive OI is still controversial (33). The effects of bisphosphonates on BMD and fractures in patients with OI type VII were inconsistent (10, 23, 29). A recent animal study suggested that sclerostin-neutralizing antibodies could increase BMD and improve the trabecular microarchitecture of Crtap^−/−^ mice (34). Anti-TGFβ treatment could improve the bone phenotype and lung abnormalities in Crtap^−/−^ mice (35). 4-Phenylbutyrate can alleviate cellular stress by restoring ER cisternae size and reducing overmodified collagen in recessive OI (32). Recently, promising strategies for OI type VII included stem cell transplantation, genetic engineering, and molecular chaperone usage (33). Elucidating the underlying molecular mechanism may lay a beneficial foundation for targeted therapies for OI type VII in the future. For instance, the correction of defects in *CRTAP* gene using different methods, such as antisense oligodeoxyribonucleotides, short interfering RNA, and hammerhead ribozymes, may have positive effects on OI type VII (33). Additionally, the use of induced pluripotent stem cells may be another interesting approach to treat OI type VII. Patient-specific induced pluripotent stem cells are a valuable tool for genetic disease treatment and are useful in drug screening (36). However, most of these approaches are still in the experimental stage, and further investigation is necessary to confirm their therapeutic benefits in OI type VII.

We conducted in-depth mechanistic research for the first time on bone specimens derived from patients with OI with the novel c.621 + 1G > A mutation in *CRTAP*. We elucidated the key pathological mechanisms by which the biallelic mutations of c.621 + 1G > A and c.1153-3C > G in *CRTAP* lead to OI type VII. However, this study still had several limitations. First, the sample size was too small to analyze the phenotypic-genotypic association in patients with OI type VII. Next, mechanistic studies were carried out in only 1 patient with compound heterozygous mutations of c.1153-3C > G and c.621 + 1G > A in *CRTAP*, which cannot represent the pathogenesis of other *CRTAP* mutations. Moreover, because it is difficult to get biopsies from participants, histological analysis was only performed on patient 2 and the age-/sex-matched healthy control participant. Although the surgical acquisition sites of the bone specimens were not identical, we were fortunate to obtain tibial samples from similar locations to minimize the potential impact of different surgical sites on histological and cellular experiments as much as possible.

Overall, the phenotype of extremely rare autosomal recessive OI type VII is characterized by early-onset recurrent fractures, severe osteoporosis, and bone deformities. The novel mutation of c.621 + 1G > A in *CRTAP* expands the genotypic spectrum of the extremely rare OI type VII. The key pathological mechanisms of OI type VII were deficiency of CRTAP in osteoblasts and reduction in prolyl 3-hydroxylation of type I collagen induced by *CRTAP* mutations, which suggests that prolyl 3-hydroxylation is important for collagen helical assembly and bone formation.

## Data Availability

All data generated or analyzed during this study are not publicly available but are available from the corresponding author at reasonable request.

## Abbreviations

25OHD: 25 hydroxyvitamin D
β-CTX: cross-linked C-telopeptide of type I collagen
ACMG/AMP: American College of Medical Genetics and Genomics and Association for Molecular Pathology
ALP: alkaline phosphatase
BMD: bone mineral density
Ca: calcium
CRTAP: cartilage-associated protein
ER: endoplasmic reticulum
FN: femoral neck
NGS: next-generation sequencing
OI: osteogenesis imperfecta
P: phosphate
P1NP: procollagen type 1 amino-terminal peptide
PUMCH: Peking Union Medical College Hospital
qPCR: quantitative PCR
